# Does Type I Interferon Limit Protective Neutrophil Responses during Pulmonary *Francisella Tularensis* Infection?

**DOI:** 10.3389/fimmu.2014.00355

**Published:** 2014-07-23

**Authors:** Yoichi Furuya, Donald Steiner, Dennis W. Metzger

**Affiliations:** ^1^Center for Immunology and Microbial Disease, Albany Medical College, Albany, NY, USA

**Keywords:** *Francisella tularensis*, mucosal immunity, type I interferon, immune evasion, neutrophil

## *Francisella* *tularensis*

Host–microorganism co-existence has enabled many pathogens to develop mechanisms to evade the immune system. One prime example is *Francisella tularensis*. This Gram-negative bacterium infects various wild animals such as rodents and rabbits, but also exists in water and soil. In rare cases, humans acquire *F. tularensis* infections through inhalation of particles from infected animals, drinking of contaminated water, ingestion of undercooked infected meat, or a bite from an infected tick or mosquito. Respiratory infection is the most deadly form of disease with a mortality rate as high as 50% if untreated. Due to its extreme virulence and ease of aerosol dissemination, it is classified as a Tier 1 bioterrorism agent by the Centers for Disease Control and Prevention. Given that inhalation of aerosolized *F. tularensis* would be the most likely route of transmission during an act of bioterrorism, recent research has shifted from intravenous/intradermal infection models to respiratory models of tularemia. Upon pulmonary infection, many cell types such as alveolar macrophages, neutrophils, and dendritic cells have been shown to harbor live *F. tularensis* (1, 2). Thus, *F. tularensis* is considered as an intracellular bacterium despite its ability to grow in a culture medium. Currently, whether phagocytes contribute to host defense or promote bacterial replication is an intense focus of biodefense research.

## Type I Interferon (IFN-I)-Mediated Suppression of Neutrophil Recruitment

*Francisella tularensis* possesses a remarkable ability to evade the host innate immune response. The first line of defense against most respiratory pathogens is the alveolar macrophage, the predominant cell type found in the airways of naïve hosts. A major role for alveolar macrophages is to efficiently eliminate invading pathogens through phagocytosis. However, *F. tularensis* has evolved a mechanism to escape macrophage killing and to replicate within phagocytes. This, together with the relatively inert properties of the *F. tularensis* LPS, renders alveolar macrophages unable to produce the cytokines and chemokines that are necessary to initiate effective immune responses during the early phases of *F. tularensis* infection (3). However, macrophage infection eventually does trigger a host immune response through bacterial recognition by cytosolic receptors, which in turn, results in type I IFN (IFN-I) production (4, 5). IFN-I signaling stimulates expression of “absent in melanoma 2” (AIM-2), a component of the inflammasome that is required for resistance against *F. tularensis* infection (5, 6). Indeed, mice deficient in AIM-2 are highly susceptible to intradermal or subcutaneous *F. tularensis* challenge (6, 7). However, a contradictory finding is that mice deficient in IFN-I receptors (IFN-IR^−/−^) exhibit substantially increased resistance to intranasal and intradermal *Francisella* infection (8, 9). This resistance suggests that the detrimental effects of IFN-I outweigh the benefits of IFN-I-mediated AIM-2 expression that is required for the inflammasome response. The detrimental effects of IFN-I were ascribed to its negative influence on expression of γδ T cells that produce IL-17 (8). Increased survival of IFN-IR^−/−^ mice following subcutaneous tularemia was closely associated with an increased IL-17 response, reduced bacterial burden, and increased influx of neutrophils into the spleen (8). Suppression of IL-17 responses and neutrophil recruitment by IFN-I has similarly been reported in other infectious disease models (10). These various findings have led to the hypothesis that IL-17-mediated neutrophil recruitment is protective against *F. tularensis* infection. Consistent with this report, IL-17A deficient mice were found to be more susceptible to intranasal *F. tularensis* infection (11–13). However, although a role for IL-17 in facilitating neutrophil responses is well established (14), during pneumonic tularemia, recruitment of CD11b^+^Ly6G/C^+^ cells was not affected by the absence of IL-17A (13). This suggests that IFN-I may limit neutrophil infiltration during pulmonary *F. tularensis* infection independently from its effects on IL-17 expression. It would be of considerable interest to assess production of neutrophil chemoattractants, such as CXCL1 and CXCL2 in the lungs of IFN-IR^−/−^ mice following intranasal *F. tularensis* infection, to elucidate the mechanisms responsible for IFN-I-mediated suppression of neutrophil recruitment.

An alternative or complementary mechanism that may be responsible for decreased neutrophil recruitment to the site of infection by IFN-I involves host cell death. Immune cells such as lymphocytes, macrophages, and neutrophils can be sensitized by IFN-I toward cell death in a mouse model of *Francisella* and *Listeria monocytogenes* infection (5, 15–17). Consistent with the findings in mice, human neutrophils with upregulated interferon-stimulated gene expression exhibit enhanced cell death following *in vitro* exposure to *Staphylococcus aureus* (15). This phenomenon, however, seems to be pathogen-specific since enhanced cell death was not observed after exposure to *Pseudomonas aeruginosa* (15). Indeed, spontaneous death of neutrophils in the absence of pathogen can be delayed by *in vitro* IFN-I treatment (1). Thus, the direct effect of IFN-I signaling on neutrophils could be context-dependent. In the case of *Francisella infection*, IFN-I signaling may promote neutrophil death and contribute to the overall decrease in neutrophil numbers at the site of infection.

## Controversial Role of Neutrophils during Pulmonary Tularemia

Neutrophils make up the first wave of phagocytic cell migration into the lungs during most pulmonary bacterial infections, including respiratory tularemia (2). However, previous studies utilizing the neutrophil depleting antibody, anti-Gr-1, have failed to show an important role for neutrophils during pulmonary *F. tularensis* infection, as assessed by bacterial burden (18). This is in striking contrast to the known importance of neutrophils in host defense against systemic tularemia (18–20). Recently, it has been proposed that neutrophils may even promote immunopathology during pulmonary tularemia. Mice deficient in metalloproteinase-9 (MMP-9), a mediator of leukocyte migration, have reduced neutrophil recruitment into the lungs following intranasal *F. tularensis* infection (21). Surprisingly, these mice exhibit better survival compared to wild-type mice following pulmonary tularemia. The interpretation from this study was that the excessive neutrophil infiltration observed in wild-type mice was detrimental to the host, but the limited recruitment of neutrophils seen in MMP-9^−/−^ mice was beneficial (21). We have recently found that near complete depletion of neutrophils using a neutrophil-specific mAb, anti-Ly6G, significantly reduced survival of intranasal *Francisella*-infected mice, an observation that is consistent with a protective role for low to moderate recruitment of neutrophils (unpublished observations). Overall, the accumulating evidence suggests that neutrophils do play a role during pneumonic tularemia, but it remains uncertain whether neutrophils exert protective or harmful effects during pneumonic tularemia. It is important to note that some of the discrepancies in results may be due to the differences in experimental conditions such as the type of *in vivo* depleting antibody, timing of antibody administration, and/or mouse strains.

## IFN-IR^−/−^ Mice as a Model to Study Protective Role of Neutrophils during Pulmonary Tularemia

**Figure 1:**
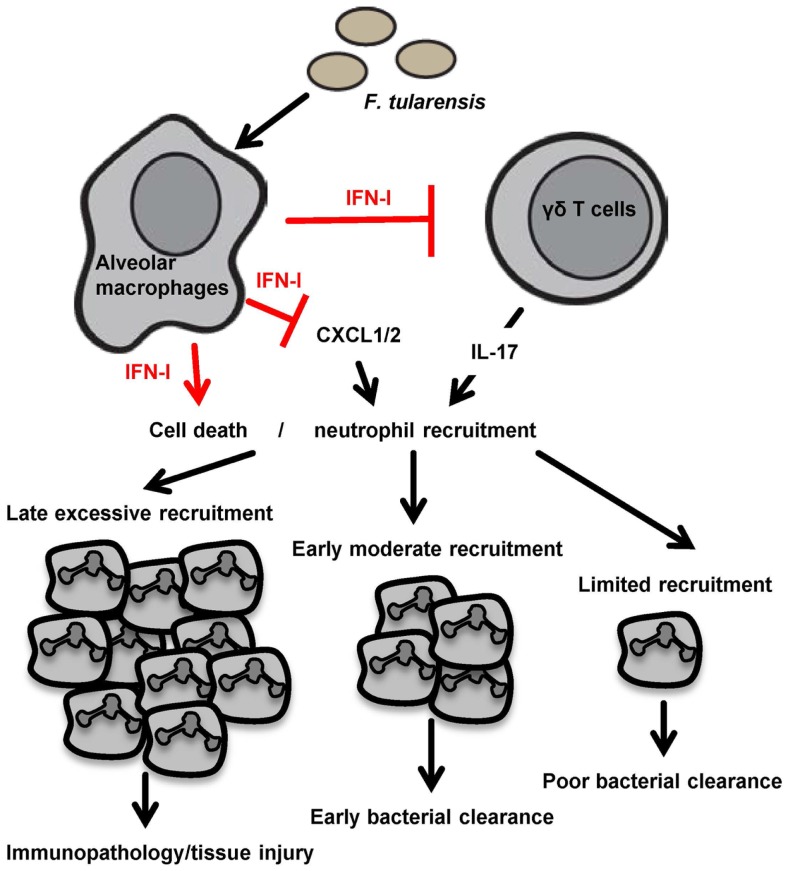
**A model depicting the proposed roles of IFN-I and neutrophils during *F. tularensis* infection is shown**. Pulmonary infection with *F. tularensis* triggers IFN-I production from innate immune cells such as alveolar macrophages. IFN-I may sensitize *F. tularensis*-infected neutrophils for cell death and contribute to an overall reduction in neutrophil numbers at the site of infection. IFN-I may also directly limit neutrophil recruitment via inhibition of IL-17 producing γδT cells or suppression of other neutrophil chemoattractants. Suppression of neutrophil recruitment may prevent tissue damage associated with excessive infiltration of neutrophils as well as lead to inadequate bacterial clearance, resulting in an unfavorable outcome. An optimal neutrophil response may require moderate recruitment at an early time point to aid in prompt clearance of bacteria without causing significant inflammation later during pulmonary tularemia.

We propose that IFN-IR^−^*^/^*^−^mice can serve as a valuable tool to better understand the role of IFN-I and neutrophils in defense of mucosal tissues against pulmonary tularemia. To the best of our knowledge, only a few studies have been performed using IFN-IR^−/−^mice in the pulmonary tularemia model. It would be of considerable interest to determine whether the absence of IFN-I signaling alters the kinetics of pulmonary neutrophil recruitment during respiratory tularemia. If so, an important question to address is whether *in vivo* depletion of neutrophils during the early versus late phases of pulmonary tularemia in IFN-IR^−/−^ mice results in a differential survival outcome. Perhaps in the absence of IFN-I signaling, there is an early influx of neutrophils into the lungs, which promotes early bacterial clearance, but later recruitment is not affected or reduced. This scenario would be consistent with the concept that neutrophils are detrimental if pulmonary infiltration is excessive (21), but their complete absence results in reduced survival. A fine balance may exist between the protective role of neutrophils during the early phase of bacterial infection versus excessive neutrophil recruitment during the recovery phase, which impedes resolution of pulmonary inflammation (depicted in Figure 1). An increased understanding of neutrophil-mediated mucosal immunity and the role IFN-I signaling in regulating recruitment of these cells to the lung may ultimately facilitate the development of novel therapeutics for protection against tularemia.

## Conflict of Interest Statement

The authors declare that the research was conducted in the absence of any commercial or financial relationships that could be construed as a potential conflict of interest.
